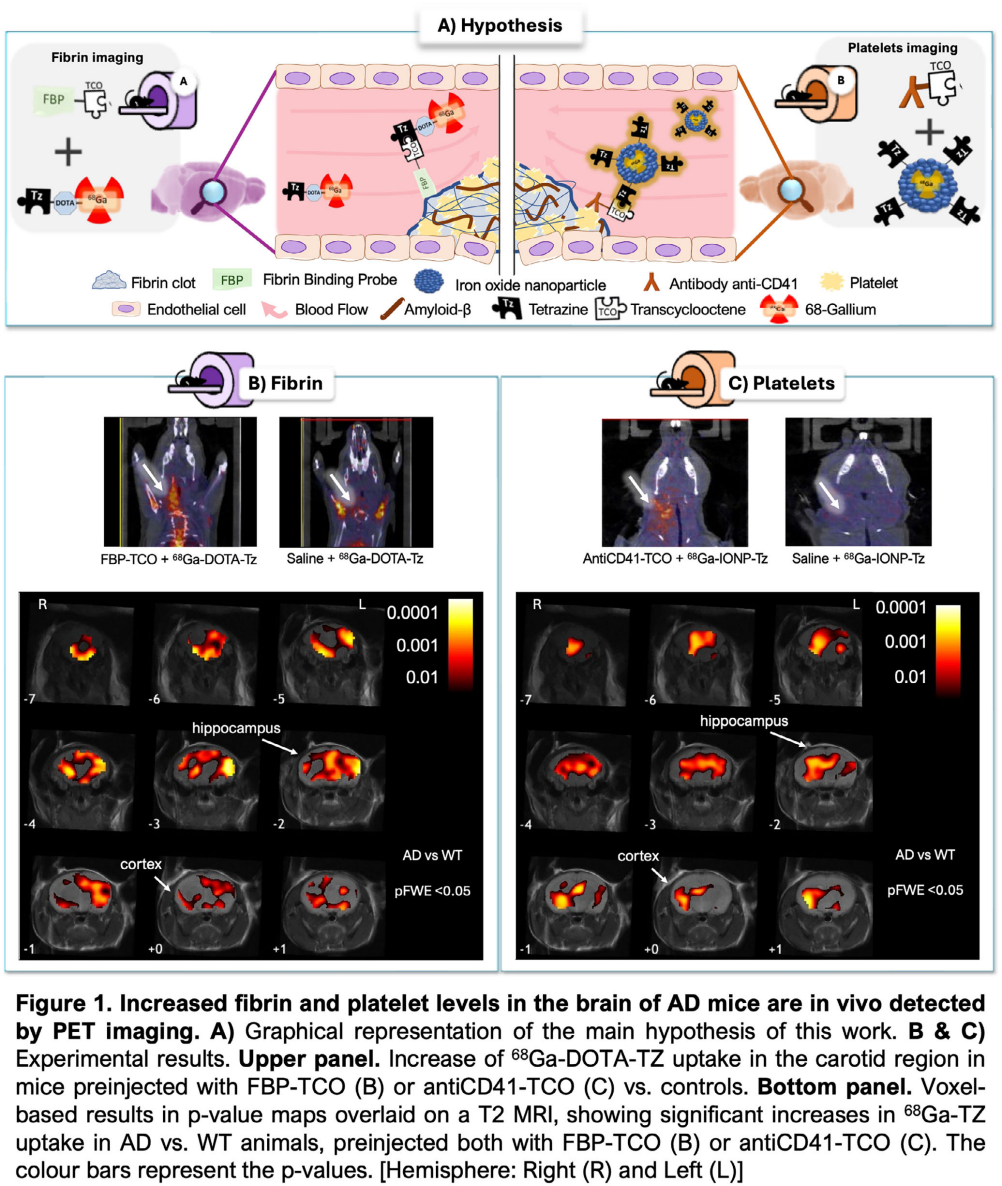# Imaging the pro‐coagulant state in Alzheimer's Disease: Detection of fibrin and platelets accumulation in the brain by PET

**DOI:** 10.1002/alz70856_100857

**Published:** 2025-12-24

**Authors:** Marta Casquero‐Veiga, Carlos Ceron, Nicolás Lamanna‐Rama, Irene Fernandez‐Nueda, Jorge Rubio‐Retama, Valentin Fuster, Maria Angeles Moro, Manuel Desco, Fernando Herranz, Beatriz Salinas, Marta Cortes‐Canteli

**Affiliations:** ^1^ Instituto de Investigación Sanitaria Fundación Jiménez Díaz (IIS‐FJD), Madrid, Madrid, Spain; ^2^ Spanish National Center for Cardiovascular Research (CNIC), Madrid, Madrid, Spain; ^3^ Consejo Superior de Investigaciones Científicas ‐ Centro Internacional de Neurociencia Cajal (CSIC ‐ CINC), Alcalá de Henares, Madrid, Spain; ^4^ Departamento de Química en Ciencias Farmacéuticas, Universidad Complutense de Madrid, Madrid, Spain; ^5^ Icahn School of Medicine at Mount Sinai, New York, NY, USA; ^6^ Spanish National Center for Cardiovascular Research (CNIC), Madrid, Spain; ^7^ Department of Bioengineering, Universidad Carlos III de Madrid (UC3M), Leganés, Madrid, Spain; ^8^ Centro de Investigación Biomédica en Red de Salud Mental (CIBERSAM), Madrid, Madrid, Spain; ^9^ Instituto de Investigación Sanitaria Gregorio Marañón (IiSGM), Madrid, Madrid, Spain; ^10^ Advanced Imaging Unit, Spanish National Center for Cardiovascular Research (CNIC), Madrid, Madrid, Spain; ^11^ Centro de Investigación Biomédica en Red de Enfermedades Respiratorias (CIBERES), Madrid, Spain; ^12^ Instituto de Química Medica (IQM), Consejo Superior de Investigaciones Científicas (CSIC), Madrid, Spain

## Abstract

**Background:**

Alzheimer's disease (AD) is a multifactorial neurodegenerative disorder with emerging evidence pointing to a pro‐thrombotic environment, supported by increased fibrin content in the brain^1^ and an unquestionable role of platelets^2^. These mechanisms contribute to hypoperfusion, blood brain barrier disruption, and neurodegeneration, occurring early in the disease but not in all patients. Early diagnosis would be invaluable to identify patients who may benefit from anticoagulant therapies^3^. To this end, we have developed two novel PET biomarkers for non‐invasive detection of brain micro‐thrombi in an AD model.

**Method:**

Vectors (fibrin binding probe ‐FBP^4^‐ and anti‐platelets antibody ‐antiCD41^5^‐) were attached to transcyclooctene (TCO), which reacts via click‐chemistry with tetrazine (TZ). TZ was used to functionalize two probes: 1) ^68^Ga‐chelator DOTA for fibrin imaging and 2) ^68^Ga‐labeled iron oxide nanoparticles for platelet imaging (Figure 1A). The experimental approach involves two steps: First, AD mice (TgCRND8) and wildtype littermates were injected with either FBP‐TCO or antiCD41‐TCO. 24 hours later, animals received ∼17 MBq IV of ^68^Ga‐Tz, and PET/CT studies were acquired with a nanoScan® PET/CT scanner (Mediso, Hungary). Brains were collected for *ex vivo* biodistribution studies. *In vivo* validation of both biomarkers was conducted in a carotid crush injury model.

**Result:**

Studies in the carotid crush injury model confirmed tracers accumulation at the damaged artery, verifying the specificity of both probes. In AD mice, ^68^Ga‐Tz uptake was elevated in the brain, independent of the target (fibrin —Figure 1B— or CD41 —Figure 1C—). These findings were confirmed by *ex vivo* studies.

**Conclusion:**

We have developed two feasible neuroimaging strategies to assess the pro‐thrombotic state in AD mice, opening a window of opportunity to ameliorate AD's progression with personalized anticoagulant treatments.

**Funding**: MCV is supported by BrightFocus Foundation, and JDC2022‐048922‐I funded by MCIN/AEI/10.13039/501100011033 and European Union NextGenerationEU/PRTR. This project is funded by the EU Joint Programme – Neurodegenerative Disease Research (JPND). CNIC is a Severo Ochoa Center of Excellence.

**References**

1. Cortes‐Canteli, et al. (2015) *Neurobiol Aging*. doi:10.1016/j.neurobiolaging.2014.10.030

2. Kucheryavykh, et al. (2017) *Brain Res Bull*. doi:10.1016/j.brainresbull.2016.11.008

3. Cortes‐Canteli, et al. (2019) *J Am Coll Cardiol*. doi:10.1016/j.jacc.2019.07.081

4. Oliveira & Caravan. (2017) *Dalton Transactions*. doi:10.1039/c7dt02634j

5. Adrover, et al. (2020) *Nanoscale*. doi:10.1039/d0nr04538a